# Relationship between parity and subclinical hypothyroidism in a large cohort of pregnant women without thyroid diseases

**DOI:** 10.1210/jendso/bvag172

**Published:** 2026-08-05

**Authors:** Laura Croce, Luca Rossi, Paolo Caccavale, Roberto Parisi, Fausta Beneventi, Camilla Bellingeri, Carlo Cappelli, Massimo Tonacchera, Flavia Magri, Mario Rotondi

**Affiliations:** Department of Internal Medicine and Therapeutics, University of Pavia, Pavia 27100, Italy; Unit of Endocrinology and Metabolism, Laboratory for Endocrine Disruptors, Istituti Clinici Scientifici Maugeri IRCCS, Pavia 27100, Italy; Department of Internal Medicine and Therapeutics, University of Pavia, Pavia 27100, Italy; Department of Internal Medicine and Therapeutics, University of Pavia, Pavia 27100, Italy; Department of Internal Medicine and Therapeutics, University of Pavia, Pavia 27100, Italy; Department of Obstetrics and Gynecology, IRCCS Foundation Policlinico San Matteo, University of Pavia, Pavia 27100, Italy; Department of Obstetrics and Gynecology, IRCCS Foundation Policlinico San Matteo, University of Pavia, Pavia 27100, Italy; Department of Clinical and Experimental Sciences, SSD Endocrinologia, University of Brescia, ASST Spedali Civili, Brescia 25100, Italy; Centro per la Diagnosi e Cura delle Neoplasie Endocrine e delle Malattie della Tiroide, University of Brescia, Brescia 25100, Italy; Department of Clinical and Experimental Medicine, Endocrine Unit, University of Pisa, Pisa 56100, Italy; Department of Internal Medicine and Therapeutics, University of Pavia, Pavia 27100, Italy; Unit of Endocrinology and Metabolism, Laboratory for Endocrine Disruptors, Istituti Clinici Scientifici Maugeri IRCCS, Pavia 27100, Italy; Department of Internal Medicine and Therapeutics, University of Pavia, Pavia 27100, Italy; Unit of Endocrinology and Metabolism, Laboratory for Endocrine Disruptors, Istituti Clinici Scientifici Maugeri IRCCS, Pavia 27100, Italy

**Keywords:** parity, thyroid, hypothyroidism, pregnancy, anti–thyroperoxidase antibodies

## Abstract

**Context:**

Increasing parity is considered a reason for screening for thyroid dysfunction during pregnancy. Nevertheless, evidence on the relationship between parity and subclinical hypothyroidism (SCH) during pregnancy is controversial and based on studies performed outside of pregnancy.

**Objective:**

To evaluate the association between parity and SCH in the first trimester of pregnancy.

**Design:**

Cross-sectional cohort study.

**Setting:**

Prenatal care outpatient services of IRCCS Policlinico San Matteo, Pavia, Italy.

**Patients:**

Pregnant women at the first antenatal visit without known thyroid disorders, personal history of autoimmune diseases, or family history of thyroid autoimmunity.

**Main Outcome Measures:**

The rates of SCH (defined as a first-trimester TSH ≥ 4 mU/L) before and after stratification for thyroid peroxidase antibody status (TPOAb)

**Results:**

The study group included 2408 women. The rates of SCH significantly increased with parity (*P* < .001) for women with 2 prior pregnancies (12/166; 7.2%) or ≥3 prior pregnancies (9/60; 15.0%) when compared with nulliparous women (51/1372; 3.7%). The rates of SCH significantly increased according to parity in TPOAb-negative women (*P* < .001) but not in TPOAb-positive women (*P* = .400). Among TPOAb-negative women, the probability of having SCH increased significantly in women who had 2 prior pregnancies (odds ratio [OR], 2.112; confidence interval [CI] 1.029-4.335, *P* = .042) and in women with ≥3 prior pregnancies (OR, 3.476; CI, 1.224-9.873; *P* = .019) when compared with nulliparous women, independently of age, body mass index, previous adverse pregnancy events and smoking.

**Conclusion:**

Higher parity is associated with a higher prevalence of SCH during pregnancy, especially in TPOAb-negative women.

Thyroid autoimmunity and dysfunction are commonly found in pregnant women. While there is general consensus regarding the need to treat maternal gestational overt hypothyroidism, the question remains open for subclinical hypothyroidism (SCH). Indeed, several studies have shown associations between SCH and a higher risk of adverse pregnancy outcomes, such as pregnancy loss (1), preeclampsia (2), gestational diabetes (3-6), small for gestational age (7), preterm birth (8), and adverse effects on offspring neurodevelopment (9, 10). Nevertheless, intervention trials have failed to show significant benefits following treatment with levothyroxine in this context (11, 12).

There is an ongoing debate regarding the screening modalities for thyroid function in pregnancy (13-16). While some authors support universal screening at the beginning of pregnancy, others support a case-finding approach. The 2017 American Thyroid Association (ATA) guidelines supported a case-finding approach based on several risk factors (17). Among these, having 2 or more prior pregnancies was considered a risk factor for thyroid dysfunction during pregnancy and thus is a criterion for thyroid function screening in a case-finding scenario. To support this recommendation, the guidelines referred to a 2014 study by Carlè et al, demonstrating that parity was a risk factor for the development of thyroid autoimmunity 1 year after delivery and not during a subsequent pregnancy (18).

In general, the role of parity as a risk factor for thyroid autoimmunity is highly debated, but most studies evaluated outcomes outside pregnancy. While some studies have suggested that parity may be a risk factor for incident thyroid dysfunction or autoimmunity after pregnancy (19, 20), other studies did not find any association (21-26). Indeed, in the latest ATA guidelines on thyroid dysfunction during pregnancy (27), parity was not included as a factor for thyroid function screening during pregnancy.

In a recent study of a cohort of thyroid peroxidase antibody (TPOAb)-negative patients specifically designed to evaluate the correlation between body mass index (BMI) and thyroid dysfunction in pregnancy, the number of previous pregnancies was not independently related to the risk of having a TSH level above 4 mU/L. Nevertheless, the study also included patients with a family history of thyroid autoimmunity and/or a personal history of other autoimmune diseases, which are relevant risk factors for thyroid dysfunction during pregnancy (28). The present study was thus specifically designed to evaluate the role of parity (considering only at-term pregnancies) as a risk factor for thyroid dysfunction in the first trimester of pregnancy in a cohort of women without previously known thyroid disease, family history of thyroid autoimmunity, or other autoimmune disorders. The principal outcome was having a TSH indicative of SCH, and patients were stratified according to the presence or absence of TPOAb positivity.

## Materials and methods

### Subjects and methods

The study protocol was already described in previous studies (29-31). The study included consecutive pregnant women at their first antenatal visit. All women were living in the area surrounding Pavia, Italy, an area characterized by a mild iodine deficiency. Inclusion criteria were (1) availability of serum TSH measurement at enrollment, (2) maternal age > 18 years, and (3) singleton pregnancy. Exclusion criteria were (1) twin pregnancies; (2) in vitro fertilization pregnancy; (3) known or clinically evident thyroid disorders, prior thyroid surgery, and/or any medication with an impact on thyroid function; (4) a family history of thyroid autoimmune diseases; (5) a personal history of nonthyroid autoimmune diseases; and (6) a history of head or neck radiation. The final study group included 2408 pregnant women. The protocol was approved by the Ethics Committee of the Fondazione IRCCS Policlinico San Matteo, Pavia, Italy (901-rcr2017i-23) and conducted in accordance with the Helsinki Declaration. All participants enrolled signed an informed consent concerning the future use of their clinical data for research purposes.

#### Data collection

All participants were evaluated during their first antenatal visit (8-10 weeks of gestation) and completed a medical questionnaire including parity, smoking status, previous miscarriage or preterm birth, preexisting diagnosis of type 1 diabetes, celiac disease, and rheumatic disease, and family history of thyroid diseases. For the aim of the present study, parity was considered as the sum of all previous pregnancies resulting in a delivery after 28 weeks of gestation, including cases of intrauterine fetal deaths. All cases of spontaneous abortion before the 28th week of gestation were not considered. Weight and height were assessed, and BMI was calculated by dividing the weight by the square of height (kg/m^2^). Blood samples were obtained in fasting condition. The serum concentrations of TSH were measured using a third-generation immunoassay, IMMULITE 2000 Systems Analyzer (Siemens Healthcare, Erlangen, Germany), employing monoclonal antibodies. In the absence of a trimester-specific reference range, we chose the reference range for TSH in the nonpregnant adult population, which is 0.4 to 4.0 mUI/L. The analytic sensitivity of the assay is 0.004 mU/L. The intra-assay coefficient of variation (CV) ranges from 3.8% to 12.5%, and the interassay CV from 4.5% to 12.5%. Serum concentrations of antithyroid peroxidase antibody (normal range ≤40 IU/mL) were measured using IMMULITE 2000 Anti-TPOAb, an enzyme-labeled, solid-phase, chemiluminescent sequential immunometric assay (Siemens Healthcare, Erlangen, Germany). The analytic sensitivity of the assay is 5.0 IU/mL. The intra-assay CV is 4.9%, and the interassay CV is 6.3% (31).

The main outcome of the study was having a TSH level ≥ 4 mU/L at the first antenatal visit.

#### Statistical analysis

Statistical analysis was performed using the SPSS software (SPSS, Inc., Evanston, IL). Continuous variables were expressed as median (25th-75th percentiles). Qualitative data were expressed as frequencies. Comparison between groups was performed using the χ^2^-test with Fisher's correction for frequencies and the Mann-Whitney (in case of 2-group comparisons) or Kruskal-Wallis (for more than 2-group comparisons) tests for continuous variables. A multivariable logistic regression model was designed including as dependent variable having a TSH≥4 U/L (yes vs no), age (years), BMI (kg/m^2^), parity (0, 1, 2 or ≥3 previous pregnancies), previous adverse pregnancy events (yes vs no) and active smoking (yes vs no) as independent predictors. A *P* < .05 was considered statistically significant.

## Results

As shown in Table 1, among the 2408 included women, 1372 (57.0%) were nulliparous, 810 women (33.6%) had 1 previous pregnancy, 166 women (6.9%) had 2 previous at-term deliveries, while 60 women (2.5%) had 3 or more previous deliveries. Specifically, 49 women (2.0%) had 3 previous deliveries, 9 women (0.4%) had 4 previous deliveries, and 2 women had 5 previous deliveries. The rate of TPOAb-positive women was 6.5%. Previous adverse pregnancy events (including miscarriage and preterm birth) were reported by 527 women (20.1%).

**Table 1:** Characteristics of the study group

Characteristic	Value
n	2408
Age, years	32.2 ± 4.9
BMI, kg/m^2^	23.1 ± 4.1
TSH, mU/L	1.60 (1.23-2.30)
TPOAb positivity, n (%)	156 (6.5)
Previous adverse pregnancy events, n (%)	527 (20.1)
Previous deliveries, n (%)	
0	1372 (57.0)
1	810 (33.6)
2	166 (6.9)
≥3	60 (2.5)

Abbreviations: BMI, body mass index; TPOAb, thyroperoxidase antibody.

**Figure 1:**
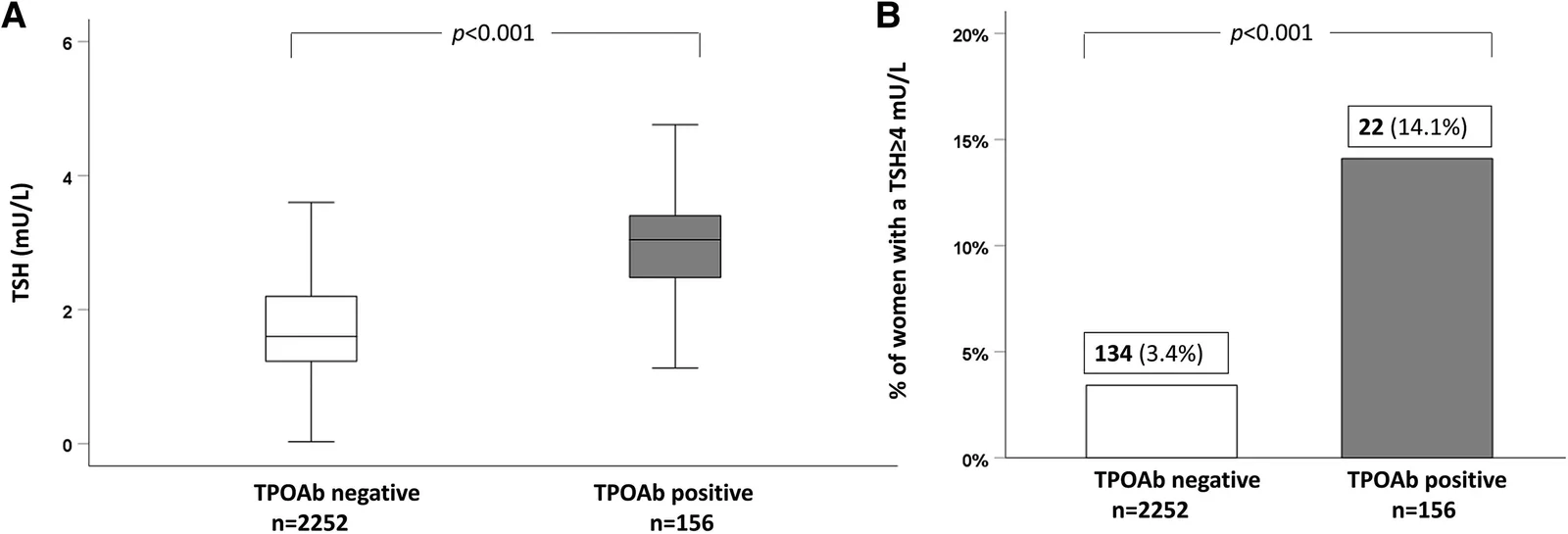
Comparison of first-trimester TSH values, both as absolute values (panel A) and rates of TSH≥4 mU/L (panel B) according to TPOAb status.

As shown in Fig. 1, TSH values were significantly higher among TPOAb-positive women (3.04 [2.46-3.40] mU/L) when compared with TPOAb-negative women (1.60 [1.22-2.20] mU/L; *P* < .001). Furthermore, the rate of women with a TSH≥4 mU/L was significantly higher among TPOAb-positive women (22/156; 14.1%) when compared with TPOAb-negative women (134/2252 women; 3.4%; *P* < .001).

### Parity status and TPOAb positivity rates and serum TSH

**Figure 2:**
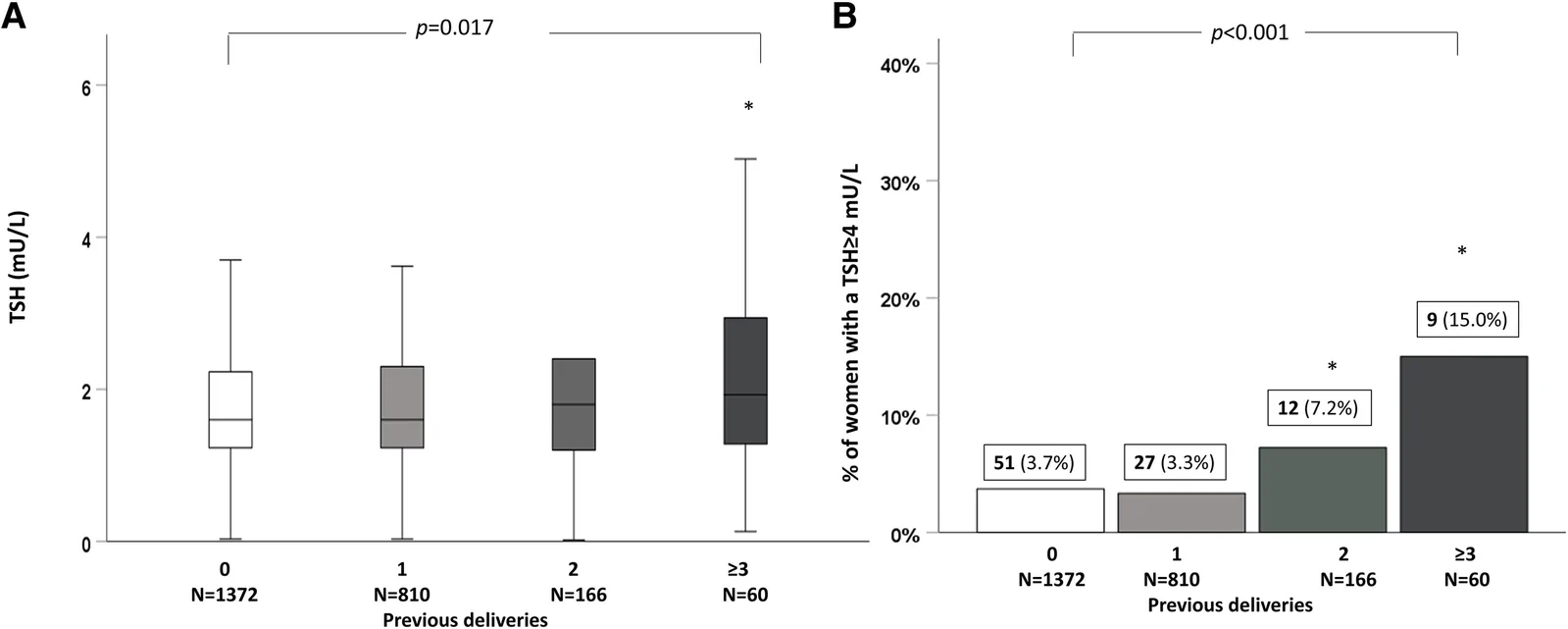
Comparison of first-trimester TSH values, both as absolute values (panel A) and rates of TSH≥4 mU/L (panel B) according to parity. * Post hoc comparison vs nulliparous women *P* < .05.

As shown in Fig. 2, serum TSH levels measured at the first postconception evaluation were higher in relation to increasing parity (H = 10.145; *P* = .017), reaching a significant difference in women who had at least 3 previous pregnancies compared with nulliparous women at post hoc analysis. The rates of women with a TSH≥4 mU/L also significantly increased with parity (*P* < .001). At post hoc analysis, percentages were significantly higher among patients who had 2 previous pregnancies (12/166 women; 7.2%) or 3 or more prior pregnancies (9/60 women; 15.0%) when compared with nulliparous women (51/1372 women; 3.7%).

**Figure 3:**
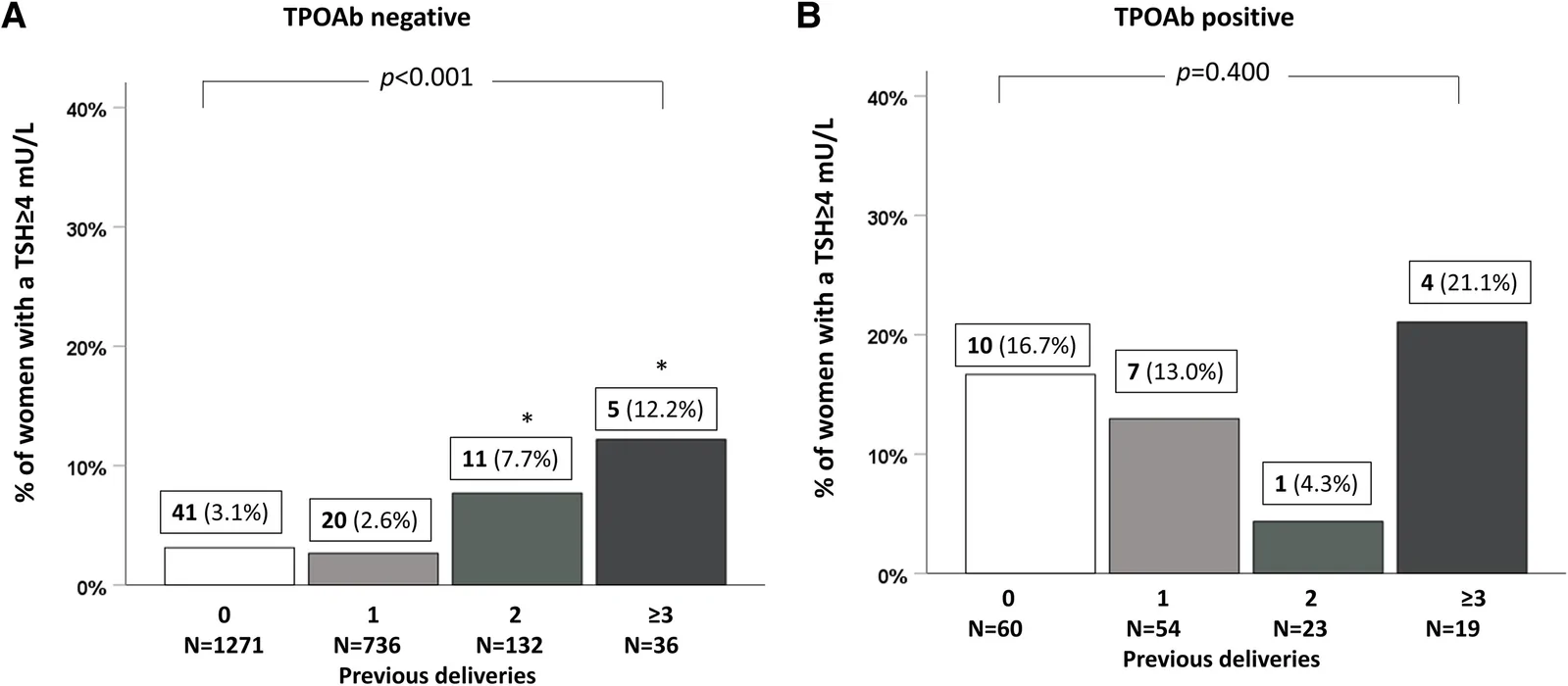
Comparison of rates of women with TSH≥4 mU/L according to parity in women with negative (panel A) and positive (panel B) TPOAb. * Post hoc comparison vs nulliparous women *P* < .05.

The stratification of patients according to TPOAb status revealed a significant difference in the rates of TSH values ≥4 mU/L in relation to parity. Indeed, as shown in Fig. 3, in women with negative TPOAb, the rates of TSH≥4 mU/L increased in a significant manner in relation to increasing parity (*P* < .001). In particular, the rates of TSH≥4 mU/L were higher among women who had at least 2 prior pregnancies (11/132 women; 7.7%) and in women who had 3 or more prior pregnancies (5/36 women; 12.2%), when compared with nulliparous women (41; 3.1%). No significant difference in the rates of TSH≥4 mU/L according to parity was observed among patients who were TPOAb-positive (*P* = .400).

To evaluate if the effect of parity observed in TPOAb-negative women was independent from other known risk factors for hypothyroidism in pregnancy, a regression model including a TSH≥4 mU/L as dependent variable and parity, maternal age, BMI, previous adverse pregnancy events, and active smoking during pregnancy as independent predictors was designed. As shown in Table 2, parity and BMI were significantly and positively associated with an increased probability of having a TSH≥4 mU/L in pregnant women with negative tests for TPOAb. In particular, the probability increased significantly in women with 2 previous pregnancies (odds ratio [OR], 2.112; 95% confidence interval [CI], 1.029-4.335; *P* = .042) and in women with 3 or more previous pregnancies (OR, 3.476; 95% CI, 1.224-9.873; *P* = .019) when compared with nulliparous women. On the contrary, no significant effect of maternal age or a history of previous adverse pregnancy events was observed. A lower probability of having a TSH ≥ 4 was observed in women who were active smokers during pregnancy (OR, 0.120; 95% CI, 0.017-0.872; *P* = .036).

**Table 2:** Results of a logistic regression analysis

	OR	95% CI for OR		*P* value
		Lower	Upper	
N of previous deliveries				**.010**
1 previous delivery vs nulliparous	0.800	0.459	1.396	.432
2 previous deliveries vs nulliparous	2.112	1.029	4.335	**.042**
≥3 previous deliveries vs nulliparous	3.476	1.224	9.873	**.019**
BMI (kg/m^2^)	1.082	1.029	1.137	**.002**
Maternal age	1.011	0.962	1.063	.669
Previous adverse pregnancy events (yes vs no)	0.761	0.407	1.423	.393
Active smoking during pregnancy (yes vs no)	0.120	0.017	0.872	**.036**

TSH ≥ 4 U/L is the dependent variable and parity. The independent predictors among TPOAb-negative women are BMI, maternal age, previous adverse pregnancy events (including preterm birth and pregnancy loss), and active smoking during pregnancy. Bold indicates *P* values lower than .05.

Abbreviations: BMI, body mass index; CI, confidence interval; OR, odds ratio; TPOAb, thyroperoxidase antibody.

## Discussion

The results of this study show that increasing parity is associated with a higher probability of having a serum level of TSH above the normal range detected at the first postconception consultation (ie, first trimester of pregnancy). In particular, the association was more evident in women with 2 or more previous pregnancies. The association was confirmed after correction for other well-known risk factors for thyroid dysfunction during pregnancy, such as maternal obesity and age (28). The association between parity and increased TSH levels appeared to differ according to TPOAb status, being evident in TPOAb-negative, but not in TPOAb-positive, women. This finding would suggest that, in the absence of more powerful risk factors, higher parity is associated with a higher likelihood of SCH at first postconception evaluation. On the other hand, given that TPOAb positivity constitutes the major risk factor for developing SCH, it seems not surprising that no significant association between parity and SCH was observed in this subgroup of patients.

The specific design of the present study included a cohort of women with no major risk factor (ie, absence of previously known thyroid diseases, positive family history of autoimmune thyroid diseases, and no concomitant autoimmune conditions). Thus, at least theoretically, these women would not have met the criteria for thyroid function screening during pregnancy in a case-finding scenario. It should be highlighted that while the 2017 ATA guidelines on thyroid dysfunction during pregnancy recommend screening women with 2 or more previous pregnancies for thyroid dysfunction when pregnant (17), this risk factor was removed from the 2026 edition (27). Indeed, given that the association between higher parity and SCH emerges only in women with no major risk factor for thyroid dysfunction, recommending screening in the general pregnant population would appear questionable.

The results of the present study appear in contrast with previous studies that failed to show any association between parity and thyroid dysfunction, although most of these studies were performed outside pregnancy (21-26, 32). Indeed, a recent single-participant meta-analysis of several maternal cohorts showed a weak association between increasing parity and risk of isolated hypothyroxinemia, while the risk of SCH was slightly lower in women with a higher number of pregnancies. Several methodological differences may explain the discrepancy between this meta-analysis and our findings. First, the study populations differed substantially. The meta-analysis included a large, heterogeneous international population, whereas our cohort consisted of women from a single mildly iodine-deficient area. Second, different definitions of thyroid dysfunction were used. Our study defined SCH as TSH ≥ 4.0 mU/L, whereas the meta-analysis used different biochemical cutoffs (TSH above the 97.5th percentile for each population included), which may have led to heterogeneous groups of patients.

Moreover, no stratification of the analysis according to TPOAb status or other major risk factors for thyroid disease was performed in the meta-analysis. Of note, the results reported herein should not be viewed as contradicting those provided by Osinga et al. Indeed, according to the results provided in a very large population, parity is not associated with an increased frequency of SCH in the general, unselected pregnant population, but this does not imply that the association is absent when a smaller but very selected low-risk population is taken into account. Indeed, in the present study, women with several major risk factors for thyroid disease, such as a family history of autoimmune thyroid disease and concomitant autoimmune disorders, were excluded, whereas the meta-analysis also included this group of patients. This may have increased the possible relative contribution of parity to thyroid dysfunction, particularly among TPOAb-negative women. Moreover, residual confounding cannot be excluded: factors such as iodine status, previous undiagnosed postpartum thyroiditis (PPT), and other population-specific characteristics may have contributed to the observed association. Finally, the 2 studies addressed different questions. While Osinga et al (32) evaluated the usefulness of parity as a screening criterion at the population level, our study examined whether parity was independently associated with thyroid dysfunction in a selected low-risk population. Therefore, the limited predictive performance of parity observed in the meta-analysis does not necessarily exclude its role as an independent factor in specific clinical settings. Our findings may reflect either a true effect of parity in selected low-risk populations or the influence of local environmental and clinical factors not fully captured in larger international analyses.

As a further point to be discussed, maternal age was not independently and significantly associated with an increased risk of having a TSH≥4 mU/L in women who were TPOAb-negative. This could be due to the fact that mean maternal age in this population was rather high. Nevertheless, these findings are in line with previous studies that failed to report any association between maternal age and risk for thyroid dysfunction (32-34). Moreover, the present results confirm the previously reported significant association between BMI and TSH levels among women who were TPOAb-negative (28).

The design of the present study does not allow us to draw firm conclusions as to the mechanisms sustaining the observed association between parity and SCH, but some considerations can be noted. Indeed, the most likely explanation may rely on the higher prevalence of iodine deficiency among women having multiple pregnancies (35, 36). It is known that at the end of pregnancy, intrathyroidal iodine stores are largely depleted due to several mechanisms, including substantial iodine loss through the fetal-placental unit and increased kidney excretion and, later in pregnancy, to the significant amount of iodine transferred from the mother to the developing fetus (37). As a result, it seems plausible to hypothesize that a higher number of previous pregnancies would result in a lower availability of iodine. Previous evidence demonstrated that the circulating level of TSH gradually increases during pregnancy in women coming from an iodine-deficient area compared with iodine-sufficient women (38). It could be argued that nowadays, most pregnant women take multivitamins during pregnancy, which in most cases contain iodine. Nevertheless, Moleti et al demonstrated that women regularly taking iodine in the 2 years before pregnancy had significantly lower TSH levels at the end of pregnancy when compared with women who were either not taking iodine supplements or had started supplementation with 150 mcg iodine at the beginning of pregnancy (39). These data would suggest that starting iodine supplementation after the detection of pregnancy may not be sufficient in women experiencing intrathyroidal iodine depletion due to previous pregnancies.

A further factor potentially contributing to the observed association stems from the following consideration. It seems reasonable to propose that a higher number of previous pregnancies has exposed the woman to a higher risk for developing PPT. Although women included in this cohort had no personal history of thyroid diseases, it should be remembered that, at least in some cases, PPT may be paucisymptomatic and/or left undiagnosed or even misdiagnosed (40). It is thus possible that pluriparous women may have experienced a PPT after one of the previous pregnancies without an established diagnosis. While this statement holds particularly true for the TPOAb-positive group, it seems worth remembering that in a significant number of cases (approximately 25-50%), PPT may occur in the absence of positive thyroid autoantibodies (41-44). This may have potentially resulted in a reduced thyroid reserve which became apparent in a subsequent pregnancy.

Although the previously mentioned mechanisms are clinically plausible, it must be highlighted that the design of the present study does not allow for specifically evaluating the role of previous episodes of PPT or the iodine status in included women. Moreover, no data regarding other possible contributing factors, such as socioeconomic factors, ethnicity, and breastfeeding history, were available in this cohort.

Lastly, since the subgroup of TPOAb-positive women was relatively small, the lack of any association between parity and SCH in this subgroup could be due to a small sample size. Larger prospective studies specifically designed to address this topic will be needed to fully demonstrate this lack of association.

In conclusion, the results of the present study demonstrate an association between higher parity and SCH at early pregnancy evaluation in women without a personal or family history of thyroid disorders and autoimmunity, particularly in those with negative TPOAb. Since thyroid dysfunction, and in particular SCH, is a recognized risk factor for maternal and neonatal complications, this information should be taken into account for programming screening policies for thyroid dysfunction during pregnancy.

## Data Availability

All datasets generated during and/or analyzed during the current study are not publicly available but are available from the corresponding author on reasonable request.
